# A case series: Association of anaphylaxis with a significant decrease in platelet levels and possible secondary risk of thrombosis

**DOI:** 10.1002/iid3.224

**Published:** 2018-04-26

**Authors:** Brian P. Peppers, Anant Vatsayan, Jignesh Dalal, Tracey Bonfield, Haig Tcheurekdjian, Robert Hostoffer

**Affiliations:** ^1^ Adult and Pediatric ACGME Osteopathic Recognized Allergy and Immunology Fellowship University Hospitals Cleveland Medical Center Cleveland Ohio; ^2^ Department of Pediatrics Hematology/Oncology and Bone Marrow Transplant Fellowship Rainbow Babies and Children's Hospital Cleveland Ohio; ^3^ Department of Immunology Case Western Reserve University Cleveland Ohio; ^4^ Allergy/Immunology Associates, Inc Mayfield Heights Ohio

**Keywords:** Anaphylaxis, idiopathic anaphylaxis, pentamidine allergy, platelet activating factor, platelet, thrombus

## Abstract

**Introduction:**

Anaphylaxis is a life threatening systemic inflammatory process that share mediators involved in the coagulation cascade. Platelet activating factor, known to increase platelet aggregation, has also been implicated as an important mediator of anaphylaxis. Although other inflammatory reactions are associated with an increased risk of thrombosis, anaphylaxis is currently not reported as one of them. Furthermore the role platelets may have in the perianaphylaxis period is not well understood. We here in present a retrospective case series of three patients that had platelet aberrations suggestive of PAF involvement and clinically significant thrombosis in close relationship with anaphylaxis.

**Objective:**

To investigate platelet response before and after anaphylaxis and indirect observation evidence of platelet activating factors involvement with possible increased risk of thrombosis.

**Methods:**

A retrospective investigation into medical records including medication administrations times, laboratory, and radiology results. Platelet levels pre‐ and post‐ anaphylaxis were statistically analyzed.

**Results:**

Case 1, a 44 year old man had an anaphylactic reaction shortly after envenomation and subsequently suffered an acute infarction with thrombus in a cerebral artery. Case 2 is a 49 year old man with idiopathic anaphylaxis who developed a deep vein thrombosis after a protracted anaphylaxis event. Case 3 involved an 18 year old female with acute myeloid leukemia was found to have a thrombus in the celiac trunk following anaphylaxis. A paired two‐tailed Wilcoxon test on the subjects pre and post anaphylactic platelet levels resulted in a overall *P* < 0.0001.

**Conclusions and Clinical Relevance:**

These three cases illustrate the potential role platelets may have in anaphylaxis and possible increased secondary risk for the development of thrombosis. Larger studies are required to determine incidence and risk factors for blood clots following anaphylaxis in order to provide management or screening recommendations.

## Introduction

Anaphylactic reactions are life threatening systemic inflammatory processes that share some mediators which are also involved in the coagulation cascade pathways. Platelet activating factor (PAF), known to increase platelet aggregation, has been implicated as an important mediator of anaphylaxis 1, 2. Although other inflammatory reactions are associated with an increased risk of thrombosis, anaphylaxis in the absence of shock is currently not reported as one of them 2, 3. The role platelets may have in the peri‐anaphylaxis period is also not well understood. We here in present a retrospective case series of three patients that had platelet aberrations suggestive of PAF involvement and clinically significant thrombosis in close relationship with anaphylaxis regardless of their shock status. We hypothesize that there is a subset of the population that are at increased risk of blood clots following anaphylaxis.

## Materials and Methods

This study was approved by University Hospitals Cleveland Medical Centers Institutional Review Board, approval number NHR‐16‐109. Patient histories, clinical event notes, laboratory and radiology results, and medication administrations times were investigated and cross referenced. GraphPad Prism 7 with confidence level of 95% was used for all statistical analysis. A paired two tailed Wilcoxon test was performed on the pre and post platelet levels due to the nonparametric distribution of the platelet levels among the three participants in the case series. A Spearman test was used on the pairing. A Kruskal‐Wallis test was performed on Case 3's platelet levels with respect to the frequency of pentamidine infusions and off pentamidine. Platelet counts are listed as “Pre‐” if they were taken the day before or earlier in the day before an attack. Platelet counts listed as “Post‐” were taken after an anaphylactic attack and were at least within 24 h of the event. Case 1 did not have a “Pre‐” platelet level. In this case the platelet level obtained within the 1st hour of the attack and was paired with a 12 h post‐event level. If more than one platelet level was obtained on the day of, but before a clinically identifiable anaphylactic attack they were matched with the platelet level of the closest circadian time frame of the following day.

## Results

### Case 1

This case was previously published and will only be reviewed briefly here 4. A 44 year old man was stung in his right knee by a flying insect. Within 20 min he developed lightheadedness, dizziness, diaphoresis, and hives. He was found by coworkers to have an ataxic gait, dysarthria, left‐sided hemiparesis, and facial droop within the hour. Treatment for anaphylaxis was given within 75 min after the envenomation(diphenhydramine, famotidine, and methylprednisolone). Epinephrine was never given. He never displayed signs of shock. The results of magnetic resonance imaging revealed an acute infarction with thrombus in the right middle cerebral artery. A cervical magnetic resonance angiography and echocardiography did not reveal any source of emboli. His platelet levels are described below (Table 1).

**Table 1:** Platelet levels before and after anaphylaxis

Entry #	Case #	Pre (10^9^/L)	Post (10^9^/L)	% Change
1	1	259	150	−42
2	2	323	256	−21
3	2	297	222	−25
4	2^a^,^b^	235	156	−34
5	2^a^,^b^	288	148	−49
6	2	148	135	−9
7	2	199	175	−12
8	2	244	220	−10
9	2^a^	389	259	−33
10	2	276	207	−25
11	2	425	274	−36
12	3	39	36	−8
13	3	36	20	−44
14	3^c^	111	88	−21
15	3	16	25	56
16	3	43	27	−37
17	3	43	39	−9

^a^ Placed on Epinephrine drip.

^b^ Entry 4: 5am, Entry 5: 1pm blood draws on the same day.

^c^ 1st pentamidine treatment after transplant with a Platelet transfusion 2 days prior.

### Case 2

The patient is a 49 year old man with psoriasis and idiopathic anaphylaxis—generalized frequent type 5 who developed an upper extremity deep vein thrombosis (DVT) after recurrent and protracted episodes of anaphylaxis without clinical signs of shock. The patient denied a family or personal history of coagulation disorders or unprovoked blood clots.

The events leading to his DVT started weeks prior when he experienced urticaria and angioedema of his face, body, and extremities without association of additional systemic symptoms. The urticaria and angioedema was initially responsive to steroids and antihistamine treatment, but within weeks became refractory and progressive. A subsequent sudden episode of respiratory distress with hypoxia resulted in an emergent crico‐thyroidotomy.

During his hospitalization subject 2 had recurrent anaphylaxis. Multiple physicians reported bilateral wheezing and poor air exchange during respiratory distress episodes. The events were unresponsive to tracheotomy hygiene and albuterol, but resolved after IM epinephrine. One incident required an epinephrine drip for 12 h. Within 24 h of an anaphylaxis episode he developed an acute occlusive left upper extremity DVT. He has had a total of four hospital admission within 1 year for anaphylaxis. During his third admission he developed an acute worsening of his chronic left upper arm DVT. With each anaphylactic attack a drop in his platelet levels were seen (Table 1).

His work‐up for a trigger of anaphylaxis has been negative to date. His tryptase levels at baseline, during protracted anaphylaxis, and an hour after anaphylaxis have never been above 9. All histamine and histamine metabolites have also been negative to date. Investigations into malignancy, mast cell disorders, autoimmune diseases have been negative to date with the exception of his psoriasis.

### Case 3

An 18 years old female undergoing chemotherapy for acute myeloid leukemia (AML) was found to be allergic to her pentamidine treatments. She developed nonocclusive thrombus involving the celiac trunk, proximal hepatic, and splenic arteries shorty after her first pentamidine administration. She originally started intravenous (IV) pentamidine after developing a nonspecific rash (without mucosal involvement) initially attributed to trimethoprim/sulfamethoxazole.

Twenty minutes into her first administration of IV pentamidine she developed severe crippling abdominal pain, emesis, diarrhea, and light headiness followed by near‐syncope. Abdominal X‐ray showed non‐obstructive gas patterns with small bowel wall thickening. After resolution of her abdominal pain she was given a platelet transfusion for thrombocytopenia (Table 1). During the platelet infusion, she developed intense abdominal pain, hypotension, and poor perfusion. Treated with intramuscular epinephrine yielded a positive response.

An abdominal CT scan showed colonic wall thickening with surrounding inflammatory changes and concern for ischemic colitis. An MR enterography revealed a nonocclusive thrombus involving the celiac trunk, proximal hepatic, and splenic arteries. The patient underwent a partial colectomy from the mid transverse colon to mid distal colon. The pathology results showed patchy transmural ischemic colitis without evidence of local thrombosis or emboli. Continued treatments of IV pentamidine every 2 weeks were noted to have immediate production of a range of symptoms mostly commonly: abdominal pain, emesis, pruritus (originally urticaria was rarely noted), diarrhea and headaches. Pentamidine allergy was established and desensitization protocol was subsequently used. Isolated urticarial flares with each desensitization was reported without any other clinical systemic involvement.

**Figure 1:**
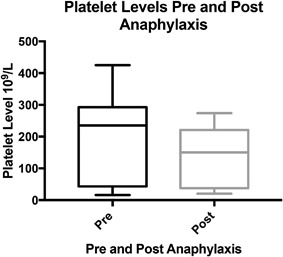
Platelet levels pre‐ and ‐post anaphylaxis. a) Wilcoxon test on a paired two tailed student *t*‐test. *P* < 0.0001.

Combined using a paired two tailed Wilcoxon test on the Pre‐ and Post‐anaphylactic platelets levels of all 3 case's showed the overall *P* < 0.0001(Figure 1). A Spearman test on the pairing had a rs value of 0.9344 with a *P* < 0.0001.

**Figure 2:**
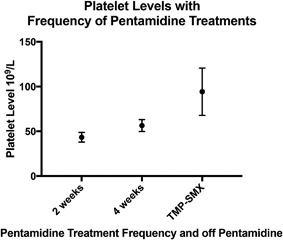
Platelet levels with frequency of pentamidine treatments. (a) Kruskal‐Wallis test, *P* < 0.0001.

For Case 3, her platelet count post BMT remained in the low 30–50's. Laboratory analysis of known specific antibodies to platelets were negative. With spacing out her pentamidine treatments to every 4 weeks her counts marginally improved to the 40–60's. Once off pentamidine, trimethoprim/sulfamethoxazole (TMP‐SMX) was restarted. Her thrombocytopenia again incrementally improved to the 80–100's. A Kruskal‐Wallis test on her platelet levels with respect to the frequency of her pentamidine infusions and off pentamidine showed a *P* < 0.0001 (Figure 2).

## Discussion

The coagulation cascade share many for the same inflammatory mediators with processes such as: sepsis, shock, asthma, allergies, and anaphylaxis. To date all of these medical ailments except for anaphylaxis is listed as being associated with increased risk of thrombosis and blood clot formation 6, 7. Platelet activating factor has been reported as a powerful stimulator of inflammatory reactions involved in both anaphylaxis and thrombus formation 1, 2, 8. The potential anaphylaxis can have on increasing an individuals risk for triggering a blood clot is unknown.

In this case series all three subjects differ greatly in the trigger of the anaphylaxis along with their respective baseline coagulation risk factors. Irrespective of the trigger or clotting risk factors, all patients suffered clinically significant thrombosis within 72 h of an anaphylactic event. For each subject this was the first occurrence of a thrombosis or blood clots in their lives. This makes the timing of the events more meaningful with simple coincidence less likely. Two of the three cases had no signs of shock during their anaphylaxis.

The association of allergies with the formation of venous thromboembolism has been explored in several studies to include: asthma, allergic rhinitis, atopic dermatitis, and increased IgE levels 7. Anaphylaxis mediated by IgE to vegetable lipoproteins specifically has been implemented in the contraction of antiphospholipid syndrome and resultant thrombosis 9, 10. In these studies thrombosis formation was seen in the peri‐anaphylactic period as well as months afterwards. The association of anaphylaxis and thrombosis formation however was not directly reported.

Case 1 had a classical IgE‐mediated anaphylactic event following venom injection by a stinging insect. Studies have already shown that venoms can trigger a decrease in factor V, VIII, and fibrinogen leading to a prothrombotic environment 11, 12. In patient 1's case he had both anaphylaxis and envenomation.

Case 2 suffered from a malignant form of idiopathic anaphylaxis—generalized frequent type 5, 13. His initial thrombus formation did have an increased risk of being seeded in his left arm secondary to a mid‐line PICC. He has subsequently had acute worsening of his chronic DVT only after an anaphylactic attack. His serial decrease in platelet levels corresponded with each attack.

Subject 3's allergic reaction to pentamidine is not clear whether her reaction was truly IgE‐mediated anaphylaxis or anaphylactoid, with the latter being favored. The spacing out of her pentamidine infusions showed modest increases in her baseline platelet levels. Once pentamidine was stopped there was an almost doubling of her previous platelet counts after 8 weeks. The exact cause of her apparent persistent mild baseline thrombocytopenia (100 to <150) is unknown. It is however reasonable given her ANOVA results on her infusion frequency and off pentamidine, that her allergy to pentamidine may have compounded her thrombocytopenia. A low level of persistent PAF stimulation, given the absence of specific antibodies to platelets on work‐up, could account for the compounded thombocyctopenia 14. Pentamidine does have an exceptionally long half‐life which would be required to achieve the persistent stimulation. Her only reaction to a platelet transfusion was within 24 h of her first pentamidine treatment and anaphylactoid reaction. This likewise can be akin to “throwing fuel on the fire” by introducing platelets into a PAF rich environment 6. This however, remains theoretical.

Studies on PAF levels and platelet activating factor acetylhydrolase (PAF‐AH) enzyme responsible for the metabolism of PAF has been reported to correlate with the severity of an anaphylactic event 15, 16. Higher levels of PAF and/or lower levels of PAF‐AH correlated with more severe anaphylactic attacks. Laboratory analysis of PAF and PAF‐AH levels are not available for clinical practice, however platelet levels are.

Transient sequestering and aggregation of platelets in the lungs and liver secondary to PAF has been reported in laboratory experiments with rabbits and other animals 2, 17. Murine studies have also studied platelets and erythrocytes response during anaphylaxis and have suggested possible participation via an allergic mechanism 18, 19. Specifically platelet's had a significant drop during anaphylaxis in the murine study by Krishnamurthy et al 18. Murine studies involving horse serum induced anaphylaxis has also noted thrombocytopenia suggesting sequestering and activating of platelets 20. To the best of our knowledge platelet's response have not been studied in anaphylaxis in humans. Reversible platelet‐endothelial interactions during inflammatory states is also well documented 6. The simple sequestering and aggregation of platelets however does not by itself indicate consumption or thrombus formation as they can return back into circulation. The overall platelet drop in relation to anaphylactic events in this case series was substantial, *P* < 0.0001.

Heparin‐induced anaphylaxis and thrombocytopenia has been reported 21. However, what triggered the thrombocytopenia was attributed to IgG antibodies to heparin‐PF4 complex. This case series suggests that there was likely two mechanisms involving the thrombocytopenia; the IgG antibodies to heparin‐PF4 complex and the actual anaphylactic episode itself. Disseminated intravascular coagulation (DIC) in extreme fatal cases of anaphylaxis has been noted in the literature 22. However, none of the three cases presented here suffered from DIC, nor were fatal.

It is well known that anaphylaxis and other inflammatory processes share common mediators with the coagulation cascade. These 3 cases illustrate the potential role platelets may play during and after IgE‐mediated anaphylaxis and non‐IgE anaphylactoid reactions in humans. Investigations into the platelets aberrations in response to and overall risk factors for blood clots following anaphylaxis are needed in order to provide conclusive recommendations. Although, the above data suggests a cautionary approach in these situations.

## Ethical Statement

This study was approved by University Hospitals Cleveland Medical Centers Institutional Review Board as non‐human research (NHR), approval number NHR‐16‐109. A waiver of consent, ethical permission, was granted secondary to the retrospective nature of this NHR study.

## Conflict of Interest

There are no conflicts of interest to report by any of the authors.
